# HIV-1 Molecular Epidemiology in Guinea-Bissau, West Africa: Origin, Demography and Migrations

**DOI:** 10.1371/journal.pone.0017025

**Published:** 2011-02-18

**Authors:** Joakim Esbjörnsson, Mattias Mild, Fredrik Månsson, Hans Norrgren, Patrik Medstrand

**Affiliations:** 1 Section of Molecular Virology, Department of Experimental Medical Science, Lund University, Lund, Sweden; 2 Department of Microbiology, Tumour and Cell Biology, Karolinska Institute, Stockholm, Sweden; 3 Department of Virology, Swedish Institute for Infectious Disease Control, Solna, Sweden; 4 Infectious Diseases Research Unit, Department of Clinical Sciences, Lund University, Malmö, Sweden; 5 Division of Infection Medicine, Department of Clinical Sciences, Lund University, Lund, Sweden; NHLS - National Health Laboratory Service - University of Pretoria, South Africa

## Abstract

The HIV-1 epidemic in West Africa has been dominated by subtype A and the recombinant form CRF02_AG. Little is known about the origins and the evolutionary history of HIV-1 in this region. We employed Maximum likelihood and Bayesian methods in combination with temporal and spatial information to reconstruct the HIV-1 subtype distribution, demographic history and migration patterns over time in Guinea-Bissau, West Africa. We found that CRF02_AG and subsubtype A3 were the dominant forms of HIV-1 in Guinea-Bissau and that they were introduced into the country on at least six different occasions between 1976 and 1981. These estimates also corresponded well with the first reported HIV-1 cases in Guinea-Bissau. Migration analyses suggested that (1) the HIV-1 epidemic started in the capital Bissau and then dispersed into more rural areas, and (2) the epidemic in Guinea-Bissau was connected to both Cameroon and Mali. This is the first study that describes the HIV-1 molecular epidemiology in a West African country by combining the results of subtype distribution with analyses of epidemic origin and epidemiological linkage between locations. The multiple introductions of HIV-1 into Guinea-Bissau, during a short time-period of five years, coincided with and were likely influenced by the major immigration wave into the country that followed the end of the independence war (1963–1974).

## Introduction

Human immunodeficiency virus type 1 (HIV-1) originated in West Central Africa via cross-species transmission from chimpanzees around the beginning of the 20^th^ century, and has since then diversified in the human population [1], [2]. Today, the most prevalent group of HIV-1 is the main (M) group which has been divided into subtypes (A–D, F–H, J–K), sub-subtypes (A1–A4, F1–F2) and 43 circulating recombinant forms (CRFs), distinguished on both the genetic level and geographic location [3].

HIV-1 mutates and recombines at extremely high rates, and the rapid generation of genetic diversity makes it possible to study the dynamics of evolutionary changes over time and to trace patterns of viral dispersal in HIV-1 epidemics [4], [5]. Coalescent theory in a phylogenetic framework has proven to be a useful tool to infer population history, and it has been used to study a variety of pathogens, including HIV-1, in different geographic regions [6], [7], [8], [9].

Little is known about the HIV-1 population dynamics and migration events that have influenced the HIV-1 epidemic in countries in West Africa. The dominating form of HIV-1 in this region is the CRF02_AG, a recombinant between the subtypes A and G [10], [11], [12], [13], [14], [15]. Most countries in West Africa reported an almost exponential increase in HIV-1 prevalence during the 1990's, reaching a steady-state level of approximately one to six percent by the end of the 1990's [16]. In Guinea-Bissau, a few cases were reported during the 1980's, and steady-state prevalence level of four to seven percent was reached by the end of the 1990's [17], [18], [19], [20], [21], [22]. Since the emergence of the AIDS epidemic, information on HIV-1 subtype distribution in Guinea-Bissau is limited to one study. Andersson *et al.* studied samples from 27 HIV-1 infected individuals collected 1994–1996 and found that 81% of the individuals were infected by CRF02_AG, 15% with subtype A, and one individual with subtype B [10].

The objective of the current study was to characterize the molecular epidemiology of HIV-1 in Guinea-Bissau, West Africa. We amplified and sequenced the HIV-1 *env* V1-V3 region (∼940 bp) from plasma samples of 82 individuals from Guinea-Bissau collected between 1993 and 2008. Maximum likelihood (ML) and Bayesian phylogenetic methods were used to reconstruct the epidemic and the demographic history of HIV-1 in Guinea-Bissau. By combining spatial and temporal information in a Bayesian phylogeographic framework we reconstructed migration events both within Guinea-Bissau and between Guinea-Bissau and other West African countries. The results of this study may have implications in understanding the epidemic potential of different HIV-1 variants, particularly in West African countries, but also in other regions of the world.

## Results

### Prevalence of HIV-1 subtypes and recombinants in Guinea-Bissau

Phylogenetic analyses showed that the most common forms of HIV-1 in Guinea-Bissau is the CRF02_AG (57%) and the subsubtype A3 (20%) (Table S1). In addition, four sequences (5%) were subtype C, one subsubtype A1 and one CRF06_cpx. Finally, 13 (16%) of the sequences were subtype A-like and clustered with long branch lengths within or close to the CRF02_AG and the A3 clusters. BootScan analyses indicated that all 13 sequences were recombinants of HIV-1 CRF02_AG and subsubtype A3. Interestingly, 12 of the 13 recombinants had a breakpoint within the C2 region of gp120. To confirm recombination, we split these sequences at their respective recombination breakpoint and constructed ML trees for each part together with the rest of the sequences from Guinea-Bissau and reference sequences. As expected, all of the identified recombinants clustered partly within the CRF02_AG cluster and partly within the subsubtype A3 cluster.

Next, we investigated differences in subtype distribution between the capital Bissau and rural Guinea-Bissau to evaluate the possibility of local epidemics related to subtype. No such differences were found: HIV-1 CRF02_AG (Bissau 55%; rural Guinea-Bissau 62%); subsubtype A3 (Bissau 23%; rural Guinea-Bissau 12%) (p = 0.35, two-tailed Fisher's exact test). Two noteworthy exceptions were found: (1) all four individuals infected with HIV-1 subtype C were sampled in the capital Bissau, and (2) there was a trend for a more profound CRF02_AG epidemic in the north-western part (10 out of 12 (83%) of the sampled sequences were CRF02_AG) as compared to the north-eastern part (three out of 8 (38%) were CRF02_AG) of rural Guinea-Bissau (p = 0.06).

### Timing of the introductions of HIV-1 CRF02_AG and A3 in Guinea-Bissau

**Figure 1:**
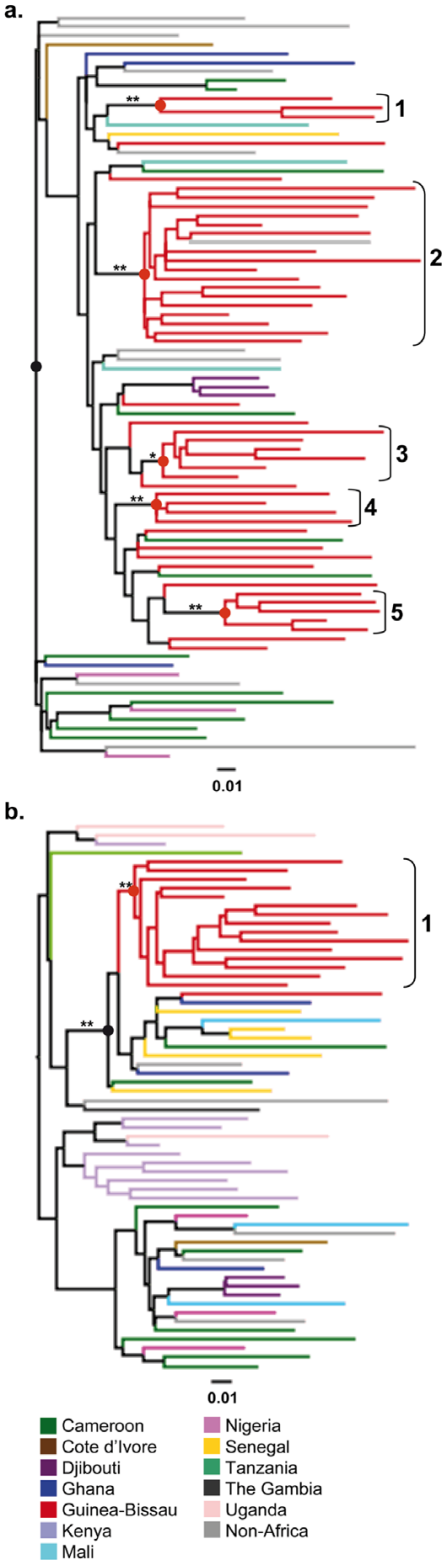
a–b. Determination of Guinea-Bissau-specific clusters in HIV-1 CRF02_AG and subsubtype A3 phylogenies. Maximum-likelihood trees were reconstructed based on the sequences from Guinea-Bissau together with reference sequences (A. CRF02_AG and B. subsubtype A3). The scale bar at the bottom of each tree represents 0.01 nucleotide substitutions per site. Asterisks along branches represent significant monophyletic clusters (**: Zero-branch-length test p-values <0.001, and posterior probabilities >95% in corresponding Bayesian analysis. *: Zero-branch-length test p-values <0.01, and posterior probabilities >90%). Statistically supported clusters are numbered according to appearance in tree and the corresponding tMRCA are marked with filled circles (red circles  =  Guinea-Bissau-specific tMRCAs, and black circles  =  the tMRCA of the CRF02_AG or subsubtype A3). The colours represent the country or geographic region representing the origin of each tip in the phylogeny.

To investigate the origin of the HIV-1 CRF02_AG and subsubtype A3 epidemics in Guinea-Bissau, we first determined different introduction events. Two sample sets with sequences from Guinea-Bissau were constructed; one with the CRF02_AG, and one with subsubtype A3 sequences. Thirty-six and 46 reference sequences from Genbank were included in the CRF02_AG and subsubtype A3 data sets, respectively. These were determined using the basic local alignment search tool (BLAST) (www.ncbi.nlm.hih.gov/BLAST) to select for the 10 most similar hits for each Guinea-Bissau-specific sequence. Details of the construction of the two sample sets can be found in the Materials and Methods section. ML and Bayesian phylogenies were reconstructed and clusters supported with zero-branch-length test (ZBLT) values of p<0.001 and posterior probabilities of >95%, were considered to be highly supported (branches denoted **, Figures 1a and b). One cluster in the CRF02_AG analysis was supported with a ZBLT value of p = 0.005 and a posterior probability value of 93% (the branch is denoted *, Figure 1a). We identified five well-supported Guinea-Bissau-specific clusters for CRF02_AG and one for subsubtype A3. We also noted that clusters three to five in the CRF02_AG phylogeny formed a cluster together with four other sequences from Guinea-Bissau and two sequences from Cameroon (Figure 1a), possibly reflecting a single common introduction. However, this large cluster was supported by neither the ZBLT nor the posterior probability. In fact, in the Bayesian maximum clade credibility tree (MCC), these three clusters did not form any potential monophyletic cluster.

Next, we estimated the timing of the identified introductions in Guinea-Bissau. The first introduction of the CRF02_AG in Guinea-Bissau was estimated to 1976 (95% highest posterior probability (HPD) 1968–1982), followed by four additional introductions during a short time period (1979–1981) (Table S2). The tMRCA for all of the CRF02_AG sequences was 1969 (95% HPD 1960–1976), and the evolutionary rate 3.80×10^−3^ substitutions site^−1^ year^−1^ (95% HPD 2.84–4.76×10^−3^). In the subsubtype A3 analysis, the tMRCA of the Guinea-Bissau cluster was estimated to 1979 (95% HPD 1960–1988). In the phylogeny of HIV-1 subsubtype A3, we identified a cluster containing both our sequences from Guinea-Bissau and some reference sequences (determined to be of subsubtype A3) that were separated from the other reference sequences (determined subtype A sequences, but not subsubtyped) (Figure 1b). Not many sequences are annotated as subsubtype A3 sequences in Genbank or the Los Alamos sequence data base, and we therefore interpreted this cluster as a true subsubtype A3 cluster. Neither the tMRCA nor the evolutionary rate for this subsubtype has been presented before. We reanalyzed this cluster and found the tMRCA of subsubtype A3 to be 1975 (95% HPD 1955–1986), and the evolutionary rate to be 3.27×10^−3^ substitutions site^−1^ year^−1^ (95% HPD 1.61–4.93×10^−3^) (Table S2).

### Detailed demographic analysis of the Guinea-Bissau HIV-1 epidemic

**Figure 2:**
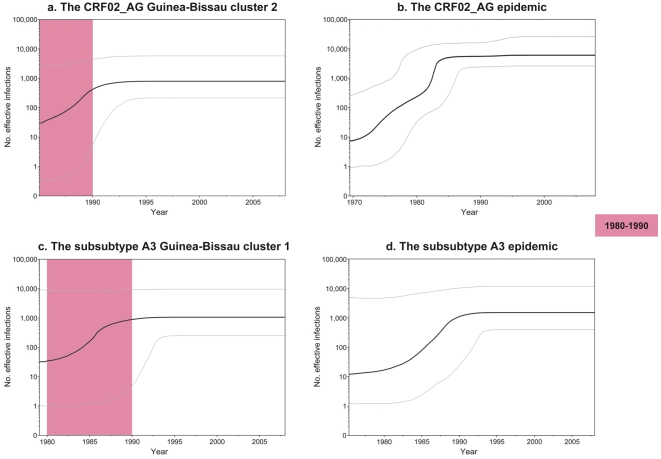
a–d. Bayesian skyline plots for the CRF02_AG and subsubtype A3 epidemics. Non-parametric median estimates of the number of effective infections over time for a. the CRF02_AG Guinea-Bissau cluster 2, b. the CRF02_AG epidemic in West Africa, c. the subsubtype A3 Guinea-Bissau cluster 1, and d. the subsubtype A3 epidemic in West Africa. All plots were based on the relaxed clock assumption. The grey lines represent the upper and lower 95% highest posterior density estimates. The pink area highlights the decade following the time period (1976–1981) of the estimated HIV-1 introductions into Guinea-Bissau.

To reconstruct the demographic history in Guinea-Bissau we focused on the two major clusters identified for the CRF02_AG (cluster 2) and the subsubtype A3 (cluster 1) epidemics, separately. The remaining clusters (CRF02_AG cluster 1 and 3–5) consisted of too few sequences to be subjected for demographic analysis. The tMRCA for cluster 2 of the CRF02_AG analysis dated back to 1976 (95% HPD 1968–1982), and contained 17 sequences from Guinea-Bissau. Demographic reconstruction of this cluster showed an exponential increase in median number of effective infections during 1985–1990 (30 to 562 effective infections), followed by an asymptotic phase towards the present (Figure 2a). A comparative demographic analysis of all Guinea-Bissau-specific CRF02_AG sequences together with the reference sequences (in total 77 sequences), showed a more pronounced demographic history (Figure 2b). An initial moderate growth phase during the 1970's was followed by a rapid exponential growth phase between 1981 and 1983, during which the number of effective infections increased from an initial median value of 350 effective infections in 1981, to a final median estimate of 4232 effective infections in 1983.

Next, we reconstructed the demographic history for subsubtype A3, both in Guinea-Bissau (cluster 1, 15 sequences), and for Guinea-Bissau specific sequences together with true subsubtype A3 reference sequences (in total 21 sequences). The tMRCA was estimated to 1979 (95% HPD 1960–1988), and the demographic analysis of subsubtype A3 in Guinea-Bissau showed a similar trend as for the CRF02_AG epidemic, with an exponential increase in the median number of effective infections between 1984 and 1987 (from 97 to 661) (Figure 2c). Inspection of the median estimates of the demographic history of the entire subsubtype A3 in West Africa suggested a slower and longer initial growth phase as compared to the CRF02_AG, starting in 1975 (at 12 effective infections), and ending in 1991 (at 1390 effective infections) (Figure 2d).

To further characterize the demographic history of HIV-1 in Guinea-Bissau, we calculated the median proportion of the current lineages that existed during 1970–1975, 1975–1980, 1980–1985, 1985–1990, and 1990–1995. The analyses showed that 79% (95% HPD 30%–94%) of the CRF02_AG lineages, and 100% (95% HPD 31%–100%) of the subsubtype A3 lineages were present in the country already in 1985 and 1990, respectively (Table 1).

**Table 1:** Bayesian estimates of median time to the most recent common ancestor, and median proportions of current lineages existing at the studied time-points.

	CRF02_AG - RC-BSP*		Subsubtype A3 - RC-BSP*	
	Median	95% HPD†	Median	95% HPD†
**tMRCA** ‡	1976	1968–1982	1979	1960–1988
**% strains in 1980**	23.4	0.2–80.8	0.6	0.6–100
**% strains in 1985**	78.7	29.8–93.6	37.5	0.6–100
**% strains in 1990**	93.6	91.5–97.9	100.0	31.3–100.0
**% strains in 1995**	93.6	91.5–95.7	93.8	93.8–100.0

In this analysis, the posterior distribution of trees was used to examine the median percentage of 2008 years strains (CRF02_AG and A3, separately) in Guinea-Bissau present in the examined years. This estimate gives an indication of changes in strain diversity during the time-periods under study.

*RC: Relaxed clock assumption. BSP10: Bayesian skyline plot used as tree prior.

† HPD: The lower and upper boundaries in 95% high posterior density interval.

‡ The estimated year of the most recent ancestor of CRF02_AG and subsubtype A3 in Guinea-Bissau, respectively. tMRCA: time to most recent common ancestor.

### Net migration rates for Guinea-Bissau between 1950 and 2010

**Figure 3:**
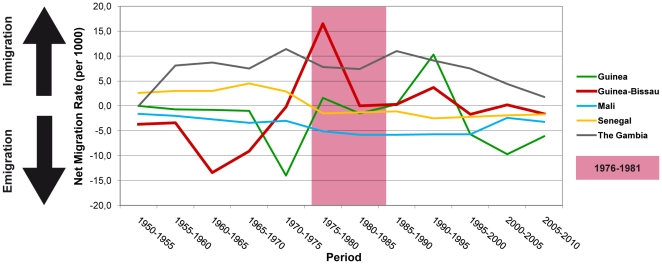
Net migration rates over 5-year intervals. Mean number of immigrating and emigrating people per 1000 individuals over different 5-year intervals from 1950 to 2010 for Guinea-Bissau and neighbouring countries (Guinea, Mali, Senegal, and The Gambia). The pink area highlights the time period of the estimated HIV-1 introductions into Guinea-Bissau. Data were obtained from the United Nations World Population Prospects (United Nations Population Division, 2010) [33].

Emigration from and immigration to Guinea-Bissau was dissected by investigating data of net migration rates from the United Nations World Population Prospects (United Nations Population Division, 2010). Two major migration waves were seen for Guinea-Bissau: (1) a net migration outflux of up to 13 of 1000 people during the mid 1960's, and (2) a net migration influx of up to 17 of 1000 people during the mid 1970's, (Figure 3). Both of these migration waves coincided with the beginning and the end of the independence war (1963–1974).

### Reconstruction of spatial dispersal patterns related to Guinea-Bissau

To reconstruct the HIV-1 CRF02_AG and A3 migration patterns over time, we removed sequences lacking information of sampling place and sampling date from the datasets used in the cluster analyses (67 and 21 sequences of CRF02_AG and subsubtype A3, respectively). For the subsubtype A3 analysis, only sequences from the identified A3 cluster were allowed. Both datasets indicated an association between geographic location and shared ancestry in the phylogenies: CRF02_AG: PS: p<0.01; AI: p<0.01); subsubtype A3: PS: p = 0.06; AI: p = 0.02.

**Figure 4:**
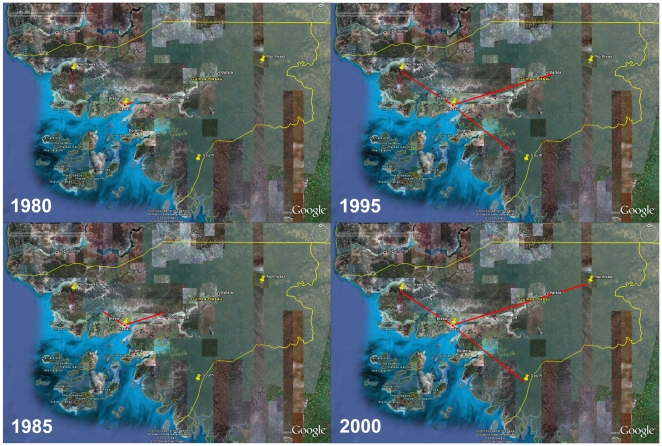
Temporal dynamics of spatial HIV-1 CRF02_AG diffusion within Guinea-Bissau. Snapshots of the HIV-1 CRF02_AG dispersal pattern within Guinea-Bissau. Lines between locations represent branches in the maximum clade credibility tree (MCC) along which well-supported location transitions occurs. The diffusion process was visualized in Google Earth (http://earth.google.com).

**Figure 5:**
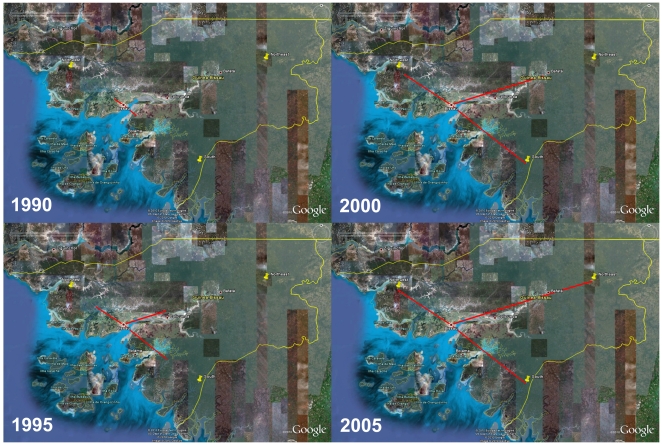
Temporal dynamics of spatial HIV-1 subsubtype A3 diffusion within Guinea-Bissau. Snapshots of the HIV-1 subsubtype A3 dispersal pattern within Guinea-Bissau. Lines between locations represent branches in the maximum clade credibility tree (MCC) along which well-supported location transitions occurs. The diffusion process was visualized in Google Earth (http://earth.google.com).

**Figure 6:**
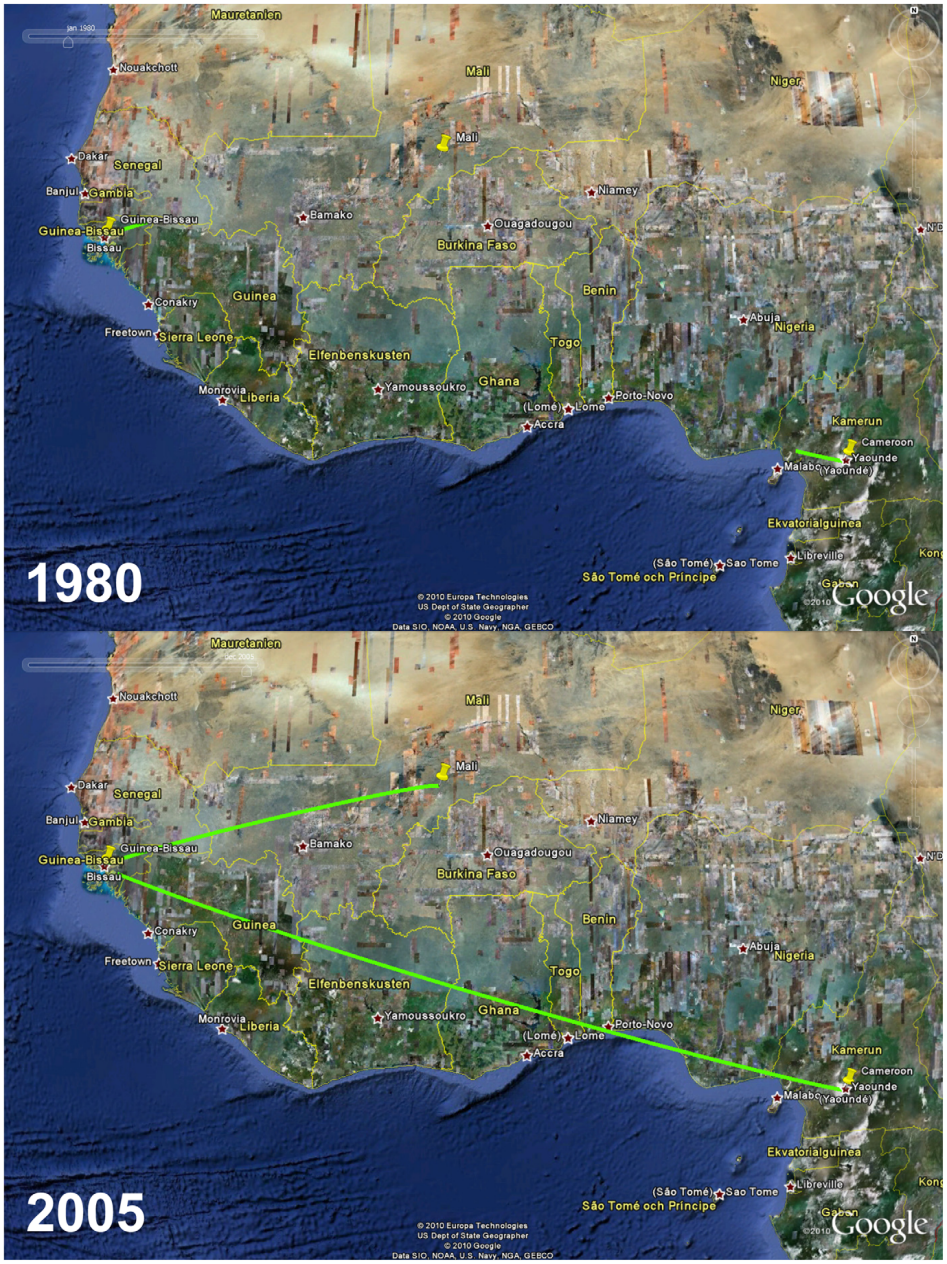
Temporal dynamics of spatial HIV-1 CRF02_AG diffusion between Guinea-Bissau and other African countries. Snapshots of the HIV-1 CRF02_AG dispersal pattern between Guinea-Bissau and other African countries. Lines between locations represent branches in the maximum clade credibility tree (MCC) along which well-supported location transitions occurs. The diffusion process was visualized in Google Earth (http://earth.google.com).

The phylogeographic analyses indicated that both the HIV-1 CRF02_AG and subsubtype A3 epidemics in Guinea-Bissau originated in the capital Bissau, from where they then dispersed out to smaller cities and villages in the countryside, probably during the 1980's and 1990's (Figures 4 and 5, Videos S1 and S2). Investigation of the diffusion patterns between Guinea-Bissau and other West African countries showed three well-supported connections for HIV-1 CRF02_AG, while none were identified for subsubtype A3. One migration event of HIV-1 CRF02_AG originated in Cameroon (1977), and reached Guinea-Bissau before 2003 (the analyzed sample was collected in 2007, but inspection of patient history data showed that the subject was HIV-1 positive in Guinea-Bissau already in 2003) (Figure 6, Video S3, Table S1). In addition, we found two separate migration events between Guinea-Bissau and Mali. Both originated in Guinea-Bissau in 1975 and 1979, respectively, and reached Mali before 2003 (Video S3).

## Discussion

HIV-1 subtype A and CRF02_AG represents approximately 27% of the worldwide HIV-1 infections, most of them prevailing in West and Central Africa [23]. Epidemiological studies from West Africa have shown that the CRF02_AG represents 50–70% of the HIV-1 infections in this region. We studied 82 HIV-1 infected individuals from Guinea-Bissau, and found that 57% were infected with CRF02_AG and 21% with subtype A. Detailed phylogenetic analysis of subtype A sequences showed that the vast majority of them (94%) belonged to the previously described subsubtype A3 [24], [25], [26]. This subsubtype was originally described in Senegal (with a prevalence of 16%), and has been shown to circulate in several countries in West Africa. We also identified 13 recombinants between HIV-1 CRF02_AG and subsubtype A3. The recombination breakpoints were located in the gp120 C2 region in 12 of the 13 recombinants. This region has previously been shown to be a recombination hot spot for subtype B [27], [28]. In addition, in the study by Meloni *et al*., where the subsubtype A3 was first described, two out of five putative HIV-1 A3 sequences were found to be recombinants between the CRF02_AG and the subsubtype A3 [24]. One of these sequences displayed a recombination breakpoint in the gp120 C2 region.

To estimate the timing of the introduction of HIV-1 in Guinea-Bissau we focused on the two dominating forms, the CRF02_AG and subsubtype A3. In the CRF02_AG phylogeny we identified one major and four minor clusters of Guinea-Bissau-specific sequences, indicating a scenario of multiple introductions into Guinea-Bissau (Figure 1a). The introduction of the major CRF02_AG cluster dated back to 1976, and was then followed by several introductions during the late 1970's and early 1980's. The timing of these introductions fits well with the results from early studies of HIV-1 seroprevalence in West Africa [22], [29]. In a study by Fultz *et al.*, one out of 440 analyzed serum samples collected in 1980 reacted in an HIV-1 radio-immunoprecipitation assay, and were considered to be potentially HIV-1 positive by the authors [22]. Kanki *et al.* analyzed 2131 serum samples, collected 1985–1987, from Guinea-Bissau and the neighbouring countries Senegal, Guinea and Mauritania and found an HIV-1 seroprevalence of 0–0.7% in the analyzed samples [29]. The results of these studies indicate an arising HIV-1 epidemic in West Africa during the early 1980's. The estimated dates of the HIV-1 CRF02_AG introductions into Guinea-Bissau are also in line with the scenario seen in the demographic analyses. The Bayesian skyline plot (BSP) analysis suggested an initial and almost asymptotic growth period of effective infections followed by a rapid exponential growth phase, starting around 1985 with a median of 30 effective infections and ending in 1990 with an almost 20-fold increase in median effective infections to 562 in Guinea-Bissau (Figure 2a). A similar pattern was seen in the analysis of the prevalence of current lineages over time. Only 23% of the current Guinea-Bissau lineages existed in the country in 1980. In 1985 the number had increased exponentially to 79%, and in 1990 almost all (94%) of the current CRF02_AG lineages were present in the country. When examining the demographic growth patterns of all CRF02_AG sequences in our dataset (representing 12 different countries) we found that the most profound increase in number of effective infections took place during 1981–1983 (with a rapid increase in median number of effective infections from 350 to 4232) (Figure 2b). Interestingly, this increase coincided with most of the introductions into Guinea-Bissau and preceded the major increase in number of effective infections in Guinea-Bissau. A closer inspection of the BSP suggested another rapid growth phase at an earlier time-point, between 1972 and 1974 (with an increase of four times in number of effective infections, from 11 to 44).

In the subsubtype A3 analysis, we identified one monophyletic cluster (1979), suggesting a major introduction followed by local epidemic spread (Figure 1b). The demographic analysis of the subsubtype A3 epidemic in Guinea-Bissau, showed similar patterns as for the CRF02_AG epidemic, with an exponential growth phase during the mid 1980's (1984–1987) (Figure 2c). Interestingly, this growth phase seemed to both predate, and to be more rapid than the one seen when analyzing all of the subsubtype A3 sequences (1985–1991) (Figure 2d).

We recognize that the demographic analyses of both the subsubtype A3 epidemic in West Africa and the local epidemics in Guinea-Bissau (CRF02_AG cluster 2 and subsubtype A3 cluster 1) are based on relatively few sequences (reflected by large HPD intervals, Figures 2a, c–d), and analyses of larger datasets are needed to confirm the results presented in this study. The rationale behind analysing well-supported clusters of country-specific sequences (instead of analysing all country-specific sequences together, regardless of clustering) is that it gives the highest probability of extracting demographic patterns specific for the country under study, without any influence of sequences from other countries. Although we stress that the demographic estimates should be interpreted with caution, it is interesting to note that the median estimates of the local demographic patterns show similar trends for both CRF02_AG and subsubtype A3 in Guinea-Bissau (Figures 2a and c). The timing of the increase in median number of effective infections also fits well with the more distinguished demographic pattern seen for the entire CRF02_AG epidemic (Figure 2b).

The phylogeographic analyses showed that HIV-1 was introduced into the urban centre (Bissau), and then spread throughout the country, a scenario that is similar to what has been reported for Cote d'Ivoire [13]. In addition, strains of the CRF02_AG epidemic, the dominant form of HIV-1 in Guinea-Bissau, started to migrate to rural areas almost 10 years before subsubtype A3, despite similar introduction dates (Figures 4 and 5). This also coincided with the exponential growth phase of the major CRF02_AG lineage (Figure 1a, cluster 2). In contrast, the subsubtype A3 epidemic grew exponentially in Bissau, and did not start to migrate to rural areas until after this phase. One might speculate that the subsubtype A3 epidemic grew in a small subpopulation not as prone to migrate as compared to the subpopulations infected with the CRF02_AG. Another possibility is that HIV-1 subsubtype A3 is less transmissible than the CRF02_AG. Competition assays in peripheral blood mononuclear cells *in vitro*, have shown that the CRF02_AG out-competes both its parental strains subtype A and G, respectively [30], [31]. The migration analyses between Guinea-Bissau and other countries in West Africa showed connections to both Cameroon and Mali (Figure 6). Since the CRF02_AG is a recombinant between the “pure” subtypes A and G, which are prevalent in central West Africa, it is reasonable to believe that the CRF02_AG arose in this area of Africa. Therefore it is not surprising to find a shared common ancestor of the Guinea-Bissau CRF02_AG epidemic in Cameroon. The connection between Guinea-Bissau and Mali is also expected since the two countries have many business interests in common. Although the migrations established in this study are important in tracing disease spread connected to Guinea-Bissau, we emphasize that these are the migrations that we can extract from the analyzed sequences. In this analysis we used all available CRF02_AG gp120 V1-V3 sequences with known sampling date and geographic location. However, it is both possible and likely that other migrations have existed in addition to those presented here and an extended analysis of a larger amount of sequences could give a more detailed picture of HIV-1 disease spread inter-connected to Guinea-Bissau.

Increased mobility and rapid urbanization is a known risk factor for acceleration of HIV transmission, creating epidemics in central cities before spreading out to more peripheral regions [32]. The estimated introductions of HIV-1 CRF02_AG in the end of the 1970's coincided with an increased immigration of people to Guinea-Bissau. According to the UN Populations Division, there was a net immigration rate of 17 per 1000 people during the period 1975–1980 into Guinea-Bissau, which corresponds in time to the repatriation of Guinea-Bissauan refugees after the declaration of independence in 1974 (Figure 3) [33]. Up to 13% of the population had fled, mainly to Senegal and Guinea during the war against the colonial Portuguese in 1963–1974 [34]. Another consequence of the war was a massive influx of population to the capital Bissau, increasing its size from 18.000 inhabitants in 1950 to 110.000 in 1974 [34].

This study is the first to investigate the molecular epidemiology and demography in a West African country, and the first to study the HIV-1 subsubtype A3 in this context. Understanding factors influencing differences in distribution, demographic history and migration patterns of different HIV-1 subtypes in West African countries are crucial to understand the epidemic potential of HIV-1 in this part of Africa.

## Materials and Methods

### Ethics

The study was approved by the Research Ethics Committee at the Karolinska Institute, Stockholm (RECKI), and the Ministries of Health and the Interior in Guinea-Bissau (MHIGB). RECKI and MHIGB also approved with a verbal consent from all study participants, due to the high rate of illiteracy in the cohort.

### Study population

Nucleotide sequences used in this study were selected from a sample set of 1,562 HIV-1 *env* V1-V3 clones derived from 156 blood plasma samples of 82 individuals, collected between 1993 and 2008 (Table S1). Seventy-seven of the samples were selected from a cohort of police officers, and five samples (DL11967-DL11971) were from a case control study [17], [19], [35], [36]. All samples had been collected in Guinea-Bissau, West Africa, and were selected based on sample availability, follow up time and disease status (patients in both asymptomatic phase and in late-stage disease are represented in the dataset). The samples used in this study are considered to be collected from individuals representative of the general population since they were all healthy by inclusion, did not display any differences in high risk-behaviour, and were all recruited from different geographical regions of Guinea-Bissau. Sample sets used in this study are further described in the section of Sample sets.

### Amplification and sequencing

Viral RNA was extracted and purified from blood plasma samples, using RNeasy Lipid Tissue Mini Kit (Qiagen, Stockholm, Sweden) with minor modifications from the manufacturer's instructions. Briefly, 200 µl of blood plasma was disrupted in 2000 µl Qiazol and 10 µg Carrier RNA was added (Qiagen). The aqueous phase was loaded onto a spin column by multiple loading steps. RNA was eluted in 40 µl of RNase-free water and treated with DNase I (Fermentas, Helsingborg, Sweden). Viral RNA was reverse transcribed using gene-specific primers, and the V1-V3 region was amplified by a nested PCR approach (The SuperScript™ III One-Step RT-PCR System with Platinum® *Taq* DNA Polymerase and Platinum® *Taq* DNA Polymerase High Fidelity, Invitrogen, Copenhagen, Denmark) according to the manufacturer's instructions using primers JE12F and V3A_R2 for one-step RT-PCR and E20A_F and JA169 for nested PCR [37], [38]. The amplified V1-V3 region of approximately 940 base pairs (nucleotides 6430 to 7374 in HXB2; GenBank accession number K03455) was cloned using the InsTAclone cloning system (Fermentas) and TOP10 cells (Invitrogen). Twelve colonies were routinely picked from each sample and the cloned fragments were amplified with Platinum® *Taq* DNA Polymerase High Fidelity (Invitrogen) using conventional M13 primers (−20 and −24). Individual clones were purified and sequenced using the BigDye Terminator v1.1 Cycle Sequencing Kit (Applied Biosystems, Stockholm, Sweden) according to the manufacturer's instructions using primers E20A_F and JA169 [37].

### Explorative sequence analysis

Sequences were assembled, and contigs were analyzed with CodonCode Aligner v1.5.2 (CodonCode Corporation, Dedham, USA). Only sequences with open reading frames were subjected to further analysis. A multiple alignment of all 1,562 sequences was created in MEGA4 using the Clustal algorithm [39], [40], [41]. The alignment was trimmed in the 3' end into complete codons, realigned at the protein level, and codon stripped to a final length of 654 bp. An explorative neighbor-joining (NJ) tree was constructed in MEGA4, using a maximum composite likelihood substitution model (default settings), to verify that all clones were of patient-specific origin (data not shown).

### PHI-test and subtype determination

Sequence recombinants violate phylogenetic inference and can lead to misinterpretations of analyzed data [42]. We used the pairwise homoplasy index (PHI) as implemented in Splitstree v4.10 to screen the 1,562 sequences for instances of intra-patient recombination events as described by Salemi *et al.* [43], [44], [45]. One hundred and eighty five potential recombinants were found and removed to a final non-recombinant dataset of 1,377 sequences representing 156 sample time-points of 82 HIV-1 infected individuals. To determine the HIV-1 subtype, one clone from each patient was randomly chosen to a sample set of 82 V1-V3 sequences and aligned with a reference sequence dataset of all major subtypes, subsubtypes and CRFs (downloaded from Los Alamos Sequence Database) in MEGA4, as described. An NJ tree was constructed in MEGA4 with complete deletion of gap positions and by using a maximum composite likelihood substitution model with heterogeneous pattern among lineages with a gamma distribution of 1.0. The phylogenetic reconstruction was bootstrapped 1,000 times to separate sequences of different subtypes. The gp120 V1-V3 region of CRF02_AG is subtype A-derived, and separation of subclusters belonging to either CRF02_AG or subtype A were not possible in this tree. To further characterize these sequences we realigned our sequences with an extended HIV-1 A-like reference sequence dataset (including the subsubtype A3 which were not included in the original dataset but has been reported to circulate in West Africa, Table S3) using PRANK_+F_ with a NJ tree (constructed in MEGA4) as guide tree [24], [46]. The PRANK_+F_ algorithm aligns sequences using phylogenetic information and has been shown to align sequences in a more evolutionary sound way, and was therefore used instead of the Clustal algorithm in this detailed analysis [46]. The alignment was manually edited and codon-stripped to a final sequence length of 681 nucleotides. A best-fitting nucleotide substitution model for the dataset was estimated using the Akaike information criterion (AIC) as implemented in Modeltest v3.6 [47]. A maximum-likelihood (ML) phylogenetic tree was constructed using the inferred model, GTR+I+G, with Garli v0.951 (www.bio.utexas.edu/faculty/antisense/garli/Garli.html) [48]. This method efficiently maximizes the tree log_e_ likelihood by using a genetic algorithm implementing the nearest neighbor interchange (NNI) and the subtree pruning regrafting (SPR) algorithms on a random starting tree to simultaneously find and optimize the topology and branch lengths [48], [49]. Support for internal branches was obtained by the ML-based zero-branch-length test (ZBLT) as implemented in PAUP* v4.0b10 [50]. We also inferred Bayesian phylogenies under the selected model using MrBayes v3.1.2 [51]. Two runs of four chains each (one cold and three heated, temp = 0.20) were run for 50 million generations, with a burn-in of 25%. The results of the two runs were combined in Logcombiner v1.5.3 [5]. Convergence was assessed by calculating the effective sampling size (ESS) using Tracer v1.5 [52]. All parameter estimates showed ESS values higher than 200. A final Bayesian majority-rule consensus tree was obtained by TreeAnnotator v1.5.3 with a burn-in of 25%.

### Recombination analysis

Five of the subtype A-like sequences fell in between the CRF02_AG and the A3 monophyletic clusters. Scattering between clusters or long branch lengths are characteristics typical for recombinant sequences [27]. To further characterize the dataset we first performed BootScan analysis to determine possible recombination breakpoints, using Simplot v3.5. NJ trees with a 200 bp moving window along the sequence alignment in 20 bp increments were constructed, and a transition/transversion ratio of 1.5 was used with a F84 model of evolution [53]. Two subgroups of A3 and CRF02_AG sequences were used as putative parental sequences, whereas a subgroup of subtype C sequences was used as outgroup. The consensus sequences of the subgroups were used in the analysis and 1,000 bootstrap replicates were generated for each analysis. Thirteen sequences from Guinea-Bissau showed mixed clustering between HIV-1 CRF02_AG and subsubtype A3 in the BootScan analysis, and displayed profound recombination breakpoints. We then examined each putative recombinant by splitting the dataset at each identified recombination breakpoint. Maximum likelihood and Bayesian trees were reconstructed as described, and sequences that clustered partly within the CRF02_AG cluster and partly within the subsubtype A3 cluster were considered to be A3/CRF02_AG recombinants.

### Sample sets for estimation of evolutionary rates and dates

Two sample sets with sequences from Guinea-Bissau were constructed; one with the CRF02_AG, and one with subsubtype A3 sequences. Since the sequences were intended to be used for estimation of evolutionary rates and dates we aimed at the widest range of collection years because a long interval of sampling years naturally provides more information about rates and divergence times than a short interval [54]. To construct a reference sequence dataset for each of the two datasets we used the basic local alignment search tool (BLAST) (www.ncbi.nlm.hih.gov/BLAST) to select for the 10 most similar hits for each Guinea-Bissau-specific sequence. Sequences sampled from the same patients were removed to a final reference dataset of 36 and 46 sequences for CRF02_AG and A3, respectively. Accession numbers of the sequences can be found in Tables S4 and S5.

### Cluster identification and estimation of evolutionary rates and dates

The two sample sets (CRF02_AG and A3) were aligned with the corresponding reference datasets using PRANK_F+_, as described in section *PHI-test and subtype determination*. The best fitting nucleotide substitution model was determined using Modeltest v3.6 (AIC). Maximum likelihood (ML) and Bayesian trees were inferred using Garli v0.951 and MrBayes v3.1.2, respectively. Statistical support for internal branches were determined by the ML-based zero-branch-length test (ZBLT), as implemented in PAUP* v4.0b10, for ML-phylogenies, and by posterior probability values for Bayesian maximum clade credibility trees (MCC) (determined by TreeAnnotator v1.5.3, as described).

Estimates of evolutionary rates (nucleotide substitutions site^−1^ year^−1^) and timing of the most recent common ancestors (tMRCA) of the datasets and clusters were performed using a Bayesian Markov Chain Monte Carlo (MCMC) approach as implemented in BEAST v1.5.3 [5]. The time span for the HIV-1 CRF02_AG and A3 datasets were 1993–2008 and 1995–2008, respectively. We used the SRD06 model implemented in BEAST v1.5.3 (a HKY85 nucleotide substitution model, with four category gamma distributed rate variation among sites, and two partitions in codon positions (1^st^+2^nd^, 3^rd^ codon)), which has been shown to be the best performing model for analysis of most viral datasets [55]. A relaxed clock with an uncorrelated lognormal distributed prior was used with the Bayesian skyline plot growth model (10 grouped intervals separated by coalescence events) as demographic model. The BSP allows the effective population size to vary between coalescence events, and does not make any restrictive prior assumptions on the demographic history. Explorative analyses of other coalescent demographic models resulted in highly similar estimates of evolutionary rates and origins (Table S2). Two independent runs of 20–100 million generations were run and samples of trees and parameter estimates were sampled every 2,000–10,000 generation. Convergence was assessed in Tracer v1.5, and the two runs were combined using Logcombiner v1.5.3 with a burn-in of 10%. A final maximum clade credibility tree was determined for each demographic model using TreeAnnotator v1.5.3. Alignments, xml-files and further details about the settings are available from the authors upon request.

### Demographic analysis and estimation of proportion of lineages

Demographic growth patterns in Guinea-Bissau were examined by analysis of the major clusters identified for HIV-1 CRF02_AG and A3, respectively. The demographic patterns of the entire CRF02_AG and subsubtype A3 epidemics were estimated based on the datasets including both sequences from Guinea-Bissau and reference sequences. We used the Bayesian skyline plot (BSP) population growth model with 10 grouped intervals as implemented in BEAST v1.5.3 [56]. Since the BSP model allows the effective population size to vary between coalescent events, the model avoids making restrictive prior assumptions about the demographic history. The MCMC analyses were run for 25–100 million generations and samples of trees and parameter estimates were sampled every 2,500–10,000 generation. Convergence was assessed in Tracer v1.5, and the two runs were combined using Logcombiner v1.5.3. A final MCC tree was determined as described.

To study the proportion of lineages existing at different time points in the Guinea-Bissau epidemic we used the program TreeStat v1.1 [57]. First, we aligned the CRF02_AG and A3 sequences of Guinea-Bissau, respectively, using PRANK_F+_ as described. The SRD06 model with the BSP population growth model with 10 grouped intervals was run for 20–100 million generations with tree and parameter sampling every 2,000–10,000 generations. In this analysis, we used the posterior distribution of trees to examine the proportion of the current CRF02_AG or A3 strains (i.e. the strains existing in Guinea-Bissau in 2008) that were present already in the examined years (1980, 1985, 1990, 1995). This estimate gives an indication of changes in strain diversity during the time-periods under study.

### Geographic information system data analysis

To investigate net migration rates we used data from the United Nations World Population Prospects (United Nations Population Division, 2010) [33]. These data are collected from various sources, including national figures on the number of immigrants and emigrants, estimates of net migration, estimates of international labor migration, and refugee stock data.

### Phylogeographic analysis

To study different migration events to and from Guinea-Bissau, as well as within Guinea-Bissau we used a recently introduced Bayesian phylogeographic model employing the Bayesian stochastic search variable selection (allowing exchange rates in the Markov model to be zero), as described by Lemey *et al*. [4]. In this methodology information about both sample time-points (temporal) and geographical locations (spatial) are used together with genetic information to infer diffusion processes among discrete locations in timed coalescence phylogenies. This makes it possible to test hypotheses about spatial dynamics and address phylogenetic uncertainty in a statistically efficient fashion. Too many geographic locations in a dataset forces the diffusion process to average over a very high state space, which can be difficult, and may lead to uncertainties in the model (P. Lemey, personal communication). To reduce the state space for the diffusion process, we removed non-African sequences from the two data sets (CRF02_AG and A3) that were used in the cluster analyses. This resulted in one dataset for the CRF02_AG (n = 68 sequences; from six different countries (locations): Guinea-Bissau, Senegal, Mali, Ghana, Nigeria and Cameroon), and one dataset for subsubtype A3 (n = 21 sequences; from four different countries (locations): Guinea-Bissau, Senegal, Mali, Cameroon). To study the diffusion process within Guinea-Bissau, Guinea-Bissau-derived sequences were further subdivided into four different locations (Central (Bissau), Northwest, Northeast, and South). Each phylogeographic analysis was run for 25–100 million generations with tree and parameter sampling every 2,500–10,000 generations in BEAST v1.5.3. Two independent runs for each setup were assessed for convergence in Tracer v1.5 with a burn-in of 10%, and combined using Logcombiner v1.5. The results were summarized in an MCC tree, and further visualized by converting the tree into a keyhole markup language (KML) file suitable for viewing in Google Earth (http://earth.google.com). Inferred states with node state probabilities >80% were considered as well supported. The KML-files were created using a java-script, as described and distributed by Lemey *et al.* and only well-supported migrations are visualized in Videos S1, S2 and S3 [4]. Alignments, xml-files and further details about the settings are available from the authors upon request.

To investigate the spatial admixture in our phylogeographic analyses and statistically measure trait associations in the phylogeny we used the parsimony score (PS) and the association index (AI), as implemented in BaTS [58]. BaTS incorporates statistical error arising from phylogenetic uncertainty by analysing the posterior distribution of trees (PDT). By comparing the PDT with a randomized null distribution, BaTS generates statistics based on the proportion of trees from the null distribution equal to, or more extreme than, the median posterior estimate of the statistics from the PDT (i.e. do closely related taxa share the same trait values to a higher extent than we would expect by chance alone?).

### Nucleotide sequence accession numbers

Nucleotide sequences were deposited in GenBank under the following accession numbers: FJ831798, FJ831808, FJ831826, FJ831843, FJ831872, FJ831878, FJ831899, FJ831907, FJ831929, FJ831955, FJ831959, FJ831971, FJ831992, FJ832012, FJ832018, FJ832041, FJ832058, FJ832064, HM745935, HM745969, HM745992, HM746011, HM746023, HM746055, HM746065, HM746110, HM746164, HM746180, HM746198, HM746236, HM746256, HM746291, HM746322, HM746349, HM746370, HM746386, HM746419, HM746448, HM746465, HM746505, HM746532, HM746543,HM746590, HQ834865-HQ834903.

## Supporting Information

Table S1 Sampling year, date of seroconversion, sample place, and HIV-1 subtype or CRF of the 82 analyzed study subjects.
(DOC)

Table S2 Estimated substitution rates and dates for the CRF02_AG and subsubtype A3 datasets by different Bayesian demographic models.
(DOC)

Table S3 Accession numbers of reference sequences representing subsubtypes A1, A2, A3 and the CRF02_AG used for detailed subtyping of the subtype A-like sequences from Guinea-Bissau.
(DOC)

Table S4 Accession numbers of reference sequences representing the CRF02_AG used for Guinea-Bissau-specific cluster identification.
(DOC)

Table S5 Accession numbers of reference sequences representing subsubtype A3 used for Guinea-Bissau-specific cluster identification.
(DOC)

Video S1 Temporal dynamics of spatial HIV-1 CRF02_AG diffusion within Guinea-Bissau.
(KML)

Video S2 Temporal dynamics of spatial HIV-1 subsubtype A3 diffusion within Guinea-Bissau.
(KML)

Video S3 Temporal dynamics of spatial HIV-1 CRF02_AG diffusion between Guinea-Bissau and other African countries.
(KML)
