# Supplementary material for: HIV-1 Molecular Epidemiology in Guinea-Bissau, West Africa: Origin, Demography and Migrations
Source: PLoS One. 2011 Feb 18;6(2):e17025. doi: 10.1371/journal.pone.0017025 (PMC3041826; doi:10.1371/journal.pone.0017025)
Supplement: Table S1 — Sampling year, date of seroconversion, sample place, and HIV-1 subtype or CRF of the 82 analyzed study subjects. (DOC) [file pone.0017025.s001.doc]

**Table S1. Sampling year, date of seroconversion, sample place, and HIV-1 subtype or CRF of the 82 analyzed study subjects.**

| **Sample*** | **Sample year** | **SC year†** | **Geography** | **Clade‡** | **Sample*** | **Sample year** | **SC year†** | **Geography** | **Clade‡** |
| --- | --- | --- | --- | --- | --- | --- | --- | --- | --- |
| DL1996H_13 | 2000 | 1990 | Bissau | CRF02_AG | DL3166F_4 | 2003 | 1999 | Bissau | A3/AG |
| DL2004F_4 | 2001 | 2001 | Northwest | CRF02_AG | DL3169F_7 | 2004 | 1999 | Bissau | CRF02_AG |
| DL2014F_1 | 1995 | 1990 | Bissau | A3 | DL3170D_5 | 1997 | 1993 | Bissau | CRF02_AG |
| DL2066G_8 | 2002 | 2000 | Bissau | CRF02_AG | DL3234J_3 | 2006 | 1999 | Bissau | A3/AG |
| DL2075I_1 | 2007 | 1998 | Bissau | CRF02_AG | DL3247F_2 | 2007 | 1999 | Bissau | CRF02_AG |
| DL2089J_9 | 2003 | 1997 | Bissau | CRF02_AG | DL3288H_5 | 2005 | NA | Northwest | CRF02_AG |
| DL2096D_7 | 1996 | 1993 | Bissau | C | DL3312C_1 | 1994 | 1993 | Northwest | CRF02_AG |
| DL2102F_1 | 1998 | 1994 | Bissau | CRF02_AG | DL3339D_12 | 2001 | 1998 | Northeast | CRF02_AG |
| DL2111E_2 | 1998 | 1994 | Bissau | CRF02_AG | DL3372I_10 | 2004 | 2000 | Northeast | A3/AG |
| DL2164F_4 | 2002 | 1997 | Bissau | A3 | DL3406J_5 | 2006 | 2000 | Northwest | A3 |
| DL2198F_3 | 1996 | 1991 | Bissau | CRF02_AG | DL3442D_8 | 2001 | 2000 | Northeast | A3/AG |
| DL2249E_8 | 1997 | 1996 | Bissau | CRF02_AG | DL3468E_10 | 2007 | 1994 | Bissau | C |
| DL2315F_4 | 2000 | 1997 | Northwest | CRF02_AG | DL3556C_9 | 1997 | 1995 | Bissau | CRF02_AG |
| DL2325J_8 | 2008 | 2000 | Bissau | CRF02_AG | DL3633G_2 | 2003 | 1999 | Bissau | CRF02_AG |
| DL2339E_4 | 2003 | 1992 | Northwest | CRF02_AG | DL3721C_3 | 1997 | 1994 | Bissau | A3/AG |
| DL2365I_3 | 2002 | 1996 | Bissau | A3 | DL3733D_9 | 2000 | 1997 | Northwest | CRF02_AG |
| DL2391B_5 | 1993 | 1991 | Bissau | CRF02_AG | DL3766D_9 | 1997 | 1994 | Bissau | C |
| DL2401M_5 | 2004 | 1996 | Bissau | CRF02_AG | DL3860H_3 | 2008 | 1995 | Bissau | A3 |
| DL2462H_4 | 2001 | 1999 | Bissau | CRF02_AG | DL3869G_3 | 2003 | NA | Bissau | A3 |
| DL2470E_5 | 2000 | 1998 | Bissau | A3 | DL3895C_2 | 1996 | 1995 | Bissau | CRF02_AG |
| DL2568E_9 | 2003 | 2003 | Bissau | A3/AG | DL3938D_3 | 1998 | 1995 | Bissau | CRF02_AG |
| DL2544F_4 | 2000 | 1999 | Northwest | CRF02_AG | DL3946E_4 | 2004 | 2001 | South | CRF02_AG |
| DL2594J_3 | 2005 | 2001 | Northeast | A3 | DL3981C_10 | 1998 | 1995 | Bissau | CRF02_AG |
| DL2596E_4 | 2000 | 1995 | South | A3 | DL4023G_7 | 2006 | 1999 | Bissau | CRF02_AG |
| DL2640I_7 | 2004 | 2003 | Bissau | CRF02_AG | DL4084F_2 | 2003 | 1999 | Bissau | A1 |
| DL2673D_4 | 1998 | 1993 | South | CRF02_AG | DL4169F_9 | 2002 | 1996 | Bissau | CRF02_AG |
| DL2713H_7 | 2007 | 2002 | Northwest | CRF02_AG | DL4214D_8 | 2002 | NA | Bissau | C |
| DL2747I_2 | 2005 | 1994 | Northwest | CRF02_AG | DL4248G_5 | 2005 | NA | Bissau | A3 |
| DL2766C_11 | 1995 | 1992 | Northwest | CRF02_AG | DL4303D_7 | 2002 | 1999 | Bissau | CRF02_AG |
| DL2829F_2 | 2006 | 1999 | Northeast | CRF06_cpx | DL4422B_4 | 2003 | NA | Northeast | CRF02_AG |
| DL2846F_8 | 2001 | 1993 | Bissau | A3 | DL4477D_9 | 2001 | NA | Northeast | CRF02_AG |
| DL2853E_2 | 1998 | 1993 | Bissau | CRF02_AG | DL4525G_2 | 2006 | 1999 | Bissau | A3 |
| DL2908G_8 | 2000 | 1997 | Bissau | CRF02_AG | DL4632E_5 | 2003 | 1997 | South | CRF02_AG |
| DL2920H_2 | 2004 | 1998 | Bissau | CRF02_AG | DL4957C_5 | 2005 | NA | Bissau | A3 |
| DL3004H_11 | 2003 | 1999 | Bissau | CRF02_AG | DL5342B_4 | 2007 | NA | South | A3/AG |
| DL3018E_10 | 2003 | 2001 | South | A3/AG | DL6324B_13 | 2007 | NA | Bissau | CRF02_AG |
| DL3037E_8 | 2005 | 1996 | Northeast | A3/AG | DL11967A_7 | 2005 | NA | Bissau | A3/AG |
| DL3039D_12 | 1998 | 1993 | Northwest | A3/AG | DL11968A_10 | 2005 | NA | Bissau | A3 |
| DL3071F_10 | 2002 | 2001 | Bissau | A3/AG | DL11969A_4 | 2005 | NA | Bissau | A3 |
| DL3087E_5 | 2001 | NA | Bissau | CRF02_AG | DL11970A_1 | 2006 | NA | Bissau | A3/AG |
| DL3098I_6 | 2007 | 2002 | Bissau | CRF02_AG | DL11971A_1 | 2006 | NA | Bissau | A3 |

*Samples identification number. The last number (i.e. _1) represents the sequence clone number.

**†**Determined seroconversion date, estimated as the date between the last HIV-1 negative sample and the first HIV-1 positive sample. NA = not applicable.

**‡**This column shows the HIV-1 subtype, CRF or other form as determined by the phylogenetic analysis.
