# Supplementary material for: HIV-1 Molecular Epidemiology in Guinea-Bissau, West Africa: Origin, Demography and Migrations
Source: PLoS One. 2011 Feb 18;6(2):e17025. doi: 10.1371/journal.pone.0017025 (PMC3041826; doi:10.1371/journal.pone.0017025)
Supplement: Table S2 — Estimated substitution rates and dates for the CRF02_AG and subsubtype A3 datasets by different Bayesian demographic models. (DOC) [file pone.0017025.s002.doc]

**Table S2**. Estimated substitution rates and dates for the CRF02_AG and subsubtype A3 datasets by different Bayesian demographic models.

| **Clade** | **Clock model*** | **Tree prior†** | **µ (10^-3^)‡** | **tMRCA§** | **Cluster 1║** | **Cluster 2║** | **Cluster 3║** | **Cluster 4║** | **Cluster 5║** |
| --- | --- | --- | --- | --- | --- | --- | --- | --- | --- |
| CRF02_AG | SC | CS | 4.27 (3.33-5.25) | 1970 (1962-1976) | 1983 (1977-1989) | 1979 (1974-1984) | 1984 (1979-1988) | 1982 (1977-1987) | 1985 (1980-1988) |
|  |  | EXP | 3.59 (2.71-4.50) | 1967 (1958-1974) | 1979 (1972-1985) | 1974 (1967-1980) | 1979 (1973-1984) | 1978 (1971-1983) | 1980 (1974-1985) |
|  |  | LOG | 3.51 (2.58-4.43) | 1966 (1957-1974) | 1979 (1972-1984) | 1974 (1966-1980) | 1978 (1971-1983) | 1977 (1969-1983) | 1980 (1973-1985) |
|  |  | BSP | 3.76 (2.87-4.68) | 1969 (1961-1976) | 1980 (1974-1985) | 1976 (1969-1981) | 1980 (1974-1984) | 1979 (1972-1984) | 1981 (1975-1985) |
|  | RC | CS | 4.37 (3.31-5.44) | 1970 (1962-1977) | 1984 (1977-1990) | 1980 (1974-1985) | 1984 (1980-1988) | 1983 (1977-1988) | 1985 (1980-1989) |
|  |  | EXP | 3.60 (2.67-4.61) | 1967 (1957-1975) | 1979 (1972-1986) | 1974 (1966-1981) | 1979 (1972-1984) | 1978 (1970-1984) | 1980 (1973-1985) |
|  |  | LOG | 3.50 (2.49-4.53) | 1967 (1955-1975) | 1979 (1971-1986) | 1974 (1964-1980) | 1978 (1970-1984) | 1977 (1968-1983) | 1980 (1972-1985) |
|  |  | BSP | 3.80 (2.84-4.76) | 1969 (1960-1976) | 1981 (1974-1986) | 1976 (1968-1982) | 1980 (1974-1984) | 1979 (1972-1984) | 1981 (1975-1985) |
| A3 | SC | CS | 3.87 (2.45-5.35) | 1978 (1966-1985) | 1982 (1972-1989) | NA^¶^ | NA^¶^ | NA^¶^ | NA^¶^ |
|  |  | EXP | 3.41 (2.10-4.90) | 1977 (1963-1985) | 1980 (1967-1987) | NA^¶^ | NA^¶^ | NA^¶^ | NA^¶^ |
|  |  | LOG | 2.92 (1.40-4.48) | 1973 (1948-1985) | 1976 (1953-1987) | NA^¶^ | NA^¶^ | NA^¶^ | NA^¶^ |
|  |  | BSP | 3.35 (1.87-4.76) | 1976 (1960-1985) | 1979 (1965-1988) | NA^¶^ | NA^¶^ | NA^¶^ | NA^¶^ |
|  | RC | CS | 3.87 (2.24-5.52) | 1977 (1962-1986) | 1983 (1970-1990) | NA^¶^ | NA^¶^ | NA^¶^ | NA^¶^ |
|  |  | EXP | 3.34 (1.88-4.96) | 1977 (1961-1986) | 1979 (1975-1988) | NA^¶^ | NA^¶^ | NA^¶^ | NA^¶^ |
|  |  | LOG | 2.78 (0.95-4.41) | 1971 (1931-1985) | 1974 (1938-1987) | NA^¶^ | NA^¶^ | NA^¶^ | NA^¶^ |
|  |  | BSP | 3.27 (1.61-4.93) | 1975 (1955-1986) | 1979 (1960-1988) | NA^¶^ | NA^¶^ | NA^¶^ | NA^¶^ |

*SC: Strict clock. RC: Relaxed clock (uncorrelated lognormal prior).

**†**Demographic model. CS: Constant size. EXP: Exponential growth. LOG: Logistic growth. BSP: Bayesian skyline plot.

**‡**µ = median substitution rate in substitutions site^-1^ year^-1^. The boundaries of the 95% higher posterior density interval are given within brackets.

**§**tMRCA = median time of the most recent common ancestor. The boundaries of the 95% higher posterior density interval are given within brackets.

**║**Dating of Guinea-Bissau specific HIV-1 CRF02_AG introductions. The boundaries of the 95% higher posterior density interval are given within brackets.

^¶^NA=not applicable.
