# Supplementary material for: HIV-1 Molecular Epidemiology in Guinea-Bissau, West Africa: Origin, Demography and Migrations
Source: PLoS One. 2011 Feb 18;6(2):e17025. doi: 10.1371/journal.pone.0017025 (PMC3041826; doi:10.1371/journal.pone.0017025)
Supplement: Table S3 — Accession numbers of reference sequences representing subsubtypes A1, A2, A3 and the CRF02_AG used for detailed subtyping of the subtype A-like sequences from Guinea-Bissau. (DOC) [file pone.0017025.s003.doc]

**Table S3. Accession numbers of reference sequences representing subsubtypes A1, A2, A3 and the CRF02_AG used for detailed subtyping of the subtype A-like sequences from Guinea-Bissau.**

AF286237

AF286238

AF286241

AY521629

AY521630

AY521631

DQ676872

AF004885

AB253421

AB253429

AY271690

L39106
