# Supplementary material for: HIV-1 Molecular Epidemiology in Guinea-Bissau, West Africa: Origin, Demography and Migrations
Source: PLoS One. 2011 Feb 18;6(2):e17025. doi: 10.1371/journal.pone.0017025 (PMC3041826; doi:10.1371/journal.pone.0017025)
Supplement: Table S4 — Accession numbers of reference sequences representing the CRF02_AG used for Guinea-Bissau-specific cluster identification. (DOC) [file pone.0017025.s004.doc]

**Table S4. Accession numbers of reference sequences representing the CRF02_AG used for Guinea-Bissau-specific cluster identification.**

AB049811

AB286863

AF063223

AF069933

AF069939

AF107770

AF184155

AF377954

AF377955

AJ251056

AJ277822

AJ286133

AJ866556

AM279352

AM279360

AM279361

AY151002

AY231152

AY231153

AY371122

AY371123

AY371126

AY371137

AY371140

AY444810

AY736840

DQ313244

DQ926899

EU480455

EU480459

EU480469

EU513187

EU786671

L22939

L23064

L39106
