# Supplementary material for: HIV-1 Molecular Epidemiology in Guinea-Bissau, West Africa: Origin, Demography and Migrations
Source: PLoS One. 2011 Feb 18;6(2):e17025. doi: 10.1371/journal.pone.0017025 (PMC3041826; doi:10.1371/journal.pone.0017025)
Supplement: Table S5 — Accession numbers of reference sequences representing subsubtype A3 used for Guinea-Bissau-specific cluster identification. (DOC) [file pone.0017025.s005.doc]

**Table S5. Accession numbers of reference sequences representing subsubtype A3 used for Guinea-Bissau-specific cluster identification.**

AB480045

AB485633

AF063223

AF069933

AF069939

AF184155

AF364109

AF457064

AF457084

AF484491

AJ286133

AJ866556

AM279347

AM279354

AM279360

AM279361

AY231152

AY371140

AY371143

AY444810

AY521629

AY521630

AY521631

AY521632

AY521633

AY669780

AY734555

AY772995

AY829207

AY905595

AY905602

DQ208445

DQ208497

EU110093

EU191612

EU480457

EU480459

EU480474

EU513189

EU853076

FJ388943

FJ866112

L22939

L23064

L39106

U15119
